# Visual and radiographic caries detection: a tailored meta-analysis for two different settings, Egypt and Germany

**DOI:** 10.1186/s12903-018-0561-z

**Published:** 2018-06-08

**Authors:** Falk Schwendicke, Karim Elhennawy, Osama El Shahawy, Reham Maher, Thais Gimenez, Fausto M. Mendes, Brian H. Willis

**Affiliations:** 1 Department of Operative and Preventive Dentistry, Charité – Universitätsmedizin Berlin, Aßmannshauser Str. 4-6, 14197 Berlin, Germany; 2 Department of Pediatric Dentistry, Cairo University, Giza, Egypt; 3 Department of Pediatric Dentistry, Cairo University, 11 el Saraya Street, Manial, Cairo, Egypt; 4 Department of Pediatric Dentistry, School of Dentistry, University of São Paulo, Av. Lineu Prestes, São Paulo, 2227 Brazil; 5 Institute of Applied Health Research, University of Birmingham, Edgbaston, Birmingham, B15 2TT UK

**Keywords:** Caries detection, Decision making, Diagnostic accuracy studies, Evidence-based dentistry, Medical informatics

## Abstract

**Background:**

Diagnostic meta-analyses on caries detection methods should assist practitioners in their daily practice. However, conventional meta-analysis estimates may be inapplicable due to differences in test conduct, applied thresholds and assessed population between settings. Our aim was to demonstrate the impact of tailored meta-analysis of visual and radiographic caries detection to different settings using setting-specific routine data.

**Methods:**

Published systematic reviews and meta-analyses on the accuracy of visual and radiographic caries detection were used. In two settings (a private practice in Germany and a public health clinic in Egypt), routine data of a total of 100 (*n* = 50/practice) consecutive 12–14 year-olds were collected. Test-positive rates of visual and radiographic detection for initial and advanced carious lesions on occlusal or proximal surfaces of molars were used to tailor meta-analyses. If prevalence data were available, these were also used for tailoring.

**Results:**

From the original reviews, 210 and 100 heterogeneous studies on visual and radiographic caries detection were included in our meta-analyses. For radiographic detection, sensitivity and specificity estimates derived from conventional and tailored meta-analysis were similar. For visual detection of advanced occlusal carious lesions, the conventional meta-analysis yielded a sensitivity and specificity (95% CI) of 64.6% (57–71) and 90.9% (88–93), whereas the tailored estimates for Egypt were 75.1% (70–81) and 84.9% (82–89), respectively, and 43.7% (37–51) and 96.5% (95–97) for Germany, respectively.

**Conclusion:**

Conventional test accuracy meta-analyses may yield aggregate estimates which are inapplicable to specific settings. Routine data may be used to produce a meta-analysis estimate which is tailored to the setting and thereby improving its applicability.

**Electronic supplementary material:**

The online version of this article (10.1186/s12903-018-0561-z) contains supplementary material, which is available to authorized users.

## Background

To detect carious lesions, dentists can use a number of methods, namely visual or visual-tactile detection, radiography, or further methods employing, for example, laser-fluorescence or near-infrared light. The accuracy of the methods is measured in terms of their sensitivity and specificity. The more sensitive methods allow the detection of early lesions, which facilitates non- or micro-invasive treatment [1, 2], but they can also lead to over-detection and overtreatment. The risks of over- and under-detection further depend on the chosen cut-off for a positive result and how the test is conducted [3–6] – does one practitioner look or search more intently for lesions than another for instance?

Theoretically, the sensitivity and specificity, and hence risks of over- and under-detection, may be determined by a Diagnostic Accuracy Study (DAS). However, DAS may vary significantly in how visual or radiographic assessment is applied, the threshold for a positive result and the type of target disorder being investigated. The test positive rate (the proportion of those who tested positive in all those tested) captures some of these features.

Moreover, DAS are often performed on different populations (many DAS on caries are performed in vitro), with different prevalence rates and patient case-mixes. This is well recognized and is sometimes described as ‘spectrum bias’ or more accurately ‘spectrum effects’ and is known to affect test performances [7]. Diagnostic meta-analysis aims to accommodate this heterogeneity [8], and provides estimates for the average sensitivity, specificity and positive or negative likelihood ratios [3, 6].

However, an average estimate may be unrepresentative of an individual dental practitioner who may use a different threshold, execute the test differently and see a population of patients which is quite different from that represented by the average sensitivity and specificity [3, 6, 9].

In response to this challenge, tailored meta-analysis (TMA) has been developed [10, 11]. Essentially the method combines routine data collected from the setting with the research data from DAS. In particular, routine data can often be collected to estimate the test positive rate and, in some cases, the prevalence. The novel aspect of the method lies in the exploitation of logical relations between the test positive rate, prevalence, sensitivity and specificity. Thus, if the first two are known it allows us to deduce the ranges of values for the sensitivity and specificity, and these may be used to tailor the selection of studies to the setting of interest for meta-analysis. Importantly, when the sensitivity and specificity for a detection method is tailored to the setting of interest this affects the positive and negative predictive values for the test in the setting, which may ultimately affect treatment decisions.

The present study used TMA to estimate the accuracy of visual and radiographic caries detection in two very different settings, Germany and Egypt. As an example, we used a population of 12- to 14-year olds, with caries detection on proximal and occlusal surfaces of permanent molars. We aimed at providing specific accuracy estimates for the two settings, hypothesizing that tailored accuracies differ between settings and from those yielded by conventional meta-analysis.

## Methods

### Study design

Conventional diagnostic meta-analyses synthesize all available data on a specific detection method. In contrast, TMA synthesizes only those data applying to a specific setting and/or particular examiners, for example with regards to test positive rate or prevalence. The present study performed TMA using three different data sources: (1) Two published systematic reviews and meta-analyses of DASs on visual and radiographic caries detection; (2) Routine data from a private practice in Germany and a public health clinic in Egypt were collected to estimate the respective test-positive rates; (3) Prevalence data for visually detected occlusal carious lesions were collected for both settings.

These are now discussed below.

### Systematic reviews

Details on the systematic review processes have been described elsewhere [3, 6]. Briefly, two reviewers searched PubMed, Embase and Cochrane Central for studies reporting accuracy data on visual [3] or radiographic detection of carious lesions [6]. The included studies reported a reference test (often histological assessment, but also other reference standards like invasive opening of the presumed lesion) to measure the performance of the index test (visual or radiographic detection). Both in vitro (the majority of studies) and clinical studies were included.

For our study, only those studies which reported sufficient data to estimate the sensitivity and specificity of the method for the proximal and/or occlusal surfaces of permanent teeth were considered. We extracted true positive, true negative, false positive and false negative numbers for each study. Data were separately extracted for studies on

1. radiographic or visual caries detection
2. detection of occlusal or proximal surfaces
3. initial and advanced lesions or only advanced lesions

Note that the definition of initial and advanced lesions was not standardized; most studies regarded cavitated lesions or those clinically and/or radiographically extended in dentin as advanced, which left non-cavitated lesions or those confined to enamel as being initial. Such dichotomization is useful, as the threshold of cavitation or dentin involvement is often used to decide between non-invasive (non-operative) and invasive (operative, restorative) treatment.

Overall, eight different subsets of data were extracted (data on visual caries detection on proximal surfaces were later discarded, as accessibility of these surfaces varied widely). As not all of the studies reported on each subset, the number of included studies varied considerably between subsets. The data used for each subset may be found in the Additional file 1.

### Routine data collection

For the present study, routine data were defined as data routinely collected on a population of patients without specific scientific purpose. Routine data thus carry the advantage that they will be available from a large range of settings, (for example claims data) and capture real-life practice. Routine data collection was approved by the local ethics committees (Charité ethics EA2 137/14; Cairo university ethics 16/7/3), and only anonymized aggregated data were used for statistical evaluation.

Over 6 months, routine examination data were collected on 50 consecutive patients aged 12–14 years old attending for a regular dental examination in each of the settings. This age was chosen, as the data were available to estimate the test positive rate and prevalence for 12-year olds in both Germany and Egypt. Patients not falling into this age band or where their data were not fully available (for example due to non-compliance with the diagnostic process) were excluded.

The data were collected by two examiners, one in each setting, and each having more than 15 years of experience in dental practice as well as experience in visual and radiographic lesion detection and evaluation.

The private practice in Germany served approximately 3000 regularly attending patients and was situated in a mid-sized town in rural Northern Germany. Regular epidemiologic surveys indicate comparatively high caries experience in this area [12, 13]. The university clinic in Egypt served approximately 250 patients per day and was situated in Giza, metropolitan region of Cairo.

Visual caries detection on molars was performed using ICDAS criteria [14], with ICDAS scores 1 and 2 being recorded as “initial lesions” and scores 3–6 as “advanced lesions”. Tooth cleaning was not routinely performed prior to the assessment, but calculus and plaque were removed if considered to be needed by the dentist. A standard dental mirror (of different manufacturers both within and between practices), a straight-ended dental probe and a 3-in-1 air/water syringe in standard dental chairs (Germany: Dentorest, Ritter, Biberach, Germany; Egypt: Belmont clesta, Takara Belmont, Tokyo, Japan) were used, with teeth being dried during examination. Cotton rolls were not regularly used during examination.

Data on visual detection were recorded only for occlusal surfaces, as visual accessibility of proximal surfaces in this age group varied greatly between patients due to different eruption status of teeth. For sealed teeth, detection was performed as far as the sealant allowed; in most cases, the surface was deemed not assessable. Restored occlusal surfaces were excluded as well. If more than one lesion was detected per occlusal surface, the worst score was recorded.

Radiographic bitewing caries detection was performed for both occlusal and proximal surfaces. In Germany, analogue Kodak Insight films (Eastman Kodak, Rochester, NY, USA) were used. Films were exposed for 0.08 s at 70kVp and 7 mA, and developed in a Dürr Dental XR 24 processing machine (Dürr, Bietigheim-Bissingen, Germany). In Egypt, Kodak Intraoral E-speed films were exposed for 0.12 s at 70kVp and 7 mA, and manually developed. Films were used in various bitewing holders (this varied across but also within settings, given this being routine application). A scoring system describing lesions in outer and inner enamel (E1, E2) and outer, middle and inner dentin (D1–3) was used. Surfaces not assessable (due to radiographic projection, or restorations or orthodontic bands present) were excluded, as were third molars. Scores were rescored into initial (E1, E2) and advanced lesions (D1-D3). Data on detection of non-occlusal and non-proximal detection were not included.

Thus, for each of the tests (visual inspection or radiography) routine data from each of the two settings were used to describe the number of lesions (initial or advanced) as a proportion of the total number of assessed (occlusal or proximal) surfaces. This enabled an interval estimate for each of the respective test positive rates.

### Estimation of the prevalence

For the two settings, we estimated the prevalence rates of visually detected initial and advanced, as well as only advanced occlusal carious lesions. For Germany, data from a city of similar size location and socioeconomic structure and a similar population of rather high risk children were used [15]. We assumed 73% of the reported lesions to be located occlusally [16]. Assuming eight occlusal molar surfaces being present and deducing the reported number of restorations (again assuming 73% being located occlusally) resulted in a tooth-level prevalence of 45% for initial and advanced, and 22% for only advanced lesions, respectively. Note that the assumption of all occlusal lesions being located on molars is a simplification and might lead to some distortion. For Egypt, data on caries experience from Cairo, the metropolis region Giza belongs to, were available [17], with 65% of reported initial and advanced lesions being situated on occlusal surfaces. Again, we assumed the lesions to be distributed among eight occlusal surfaces, which resulted in a tooth-level prevalence of 12.9% for all (initial and advanced) and 8.1% for only advanced lesions, respectively.

### Statistical analysis

Willis and Hyde [10, 11] have previously demonstrated that if the test positive rate is known this constrains the region of values that the sensitivity and specificity take in receiver operating characteristic (ROC) space. This region is constrained further if the prevalence is also known. Thus, an *applicable region* for the test sensitivity and specificity may be derived based on the test positive rate and prevalence. In essence, it represents the feasible set of (sensitivity, specificity) pairs for the test given the information we have collected on the test positive rate and prevalence. Furthermore since the test positive rate and prevalence vary with the test (visual or radiography), target disorder (all lesions or only advanced lesions) and setting (Germany and Egypt) the applicable region is particular to the respective combination of these.

After deriving the applicable region, each study is compared with this region by first deriving the maximum likelihood estimate (MLE) for the sensitivity and specificity subject to it being constrained to lie in the applicable region [10, 11]. This is then compared with the observed sensitivity and specificity and low probability studies (*p* < 0.025) are excluded. The resulting tailored set of studies is then aggregated using a bivariate random effects model to derive estimates for the sensitivity and specificity [18] - this is the TMA estimate.

The test positive rate ranges used to derive the applicable region in the TMA corresponded to the 99% confidence intervals derived from the routine data collected from each of the settings. The ranges used for the prevalence rates were based on a plausible interval that contained the point prevalence estimates derived for the setting (Table 1).

**Table 1:** Test positive rate and prevalence rates used in tailored meta-analysis

	Egypt				Germany			
	Test positive rate		Prevalence		Test positive rate		Prevalence	
	Lower limit	Upper limit	Lower limit	Upper limit	Lower limit	Upper limit	Lower limit	Upper limit
Visual detection								
advanced occlusal	0.20	0.33	0.01	0.15	0.05	0.26	0.15	0.30
all occlusal	0.30	0.44	0.05	0.20	0.13	0.28	0.35	0.55
Radiographic detection								
advanced occlusal	0.14	0.26	0.09	0.29	0.06	0.18	0.01	0.21
all occlusal	0.14	0.26	0.09	0.29	0.06	0.19	0.01	0.21
advanced proximal	0.02	0.07			0.03	0.08		
all proximal	0.03	0.08			0.07	0.14		

The lower and upper limits for the test positive rates are 99% confidence interval limits derived from the routine data. The lower and upper limits for the prevalence rates are plausible limits based on a priori point estimates

There is no reliable statistical method for estimating the level of heterogeneity when using bivariate measures (sensitivity, specificity). However, the ROC plot provides a visual representation of the dispersion of studies which is indicative of the level of heterogeneity.

## Results

**Fig. 1:**
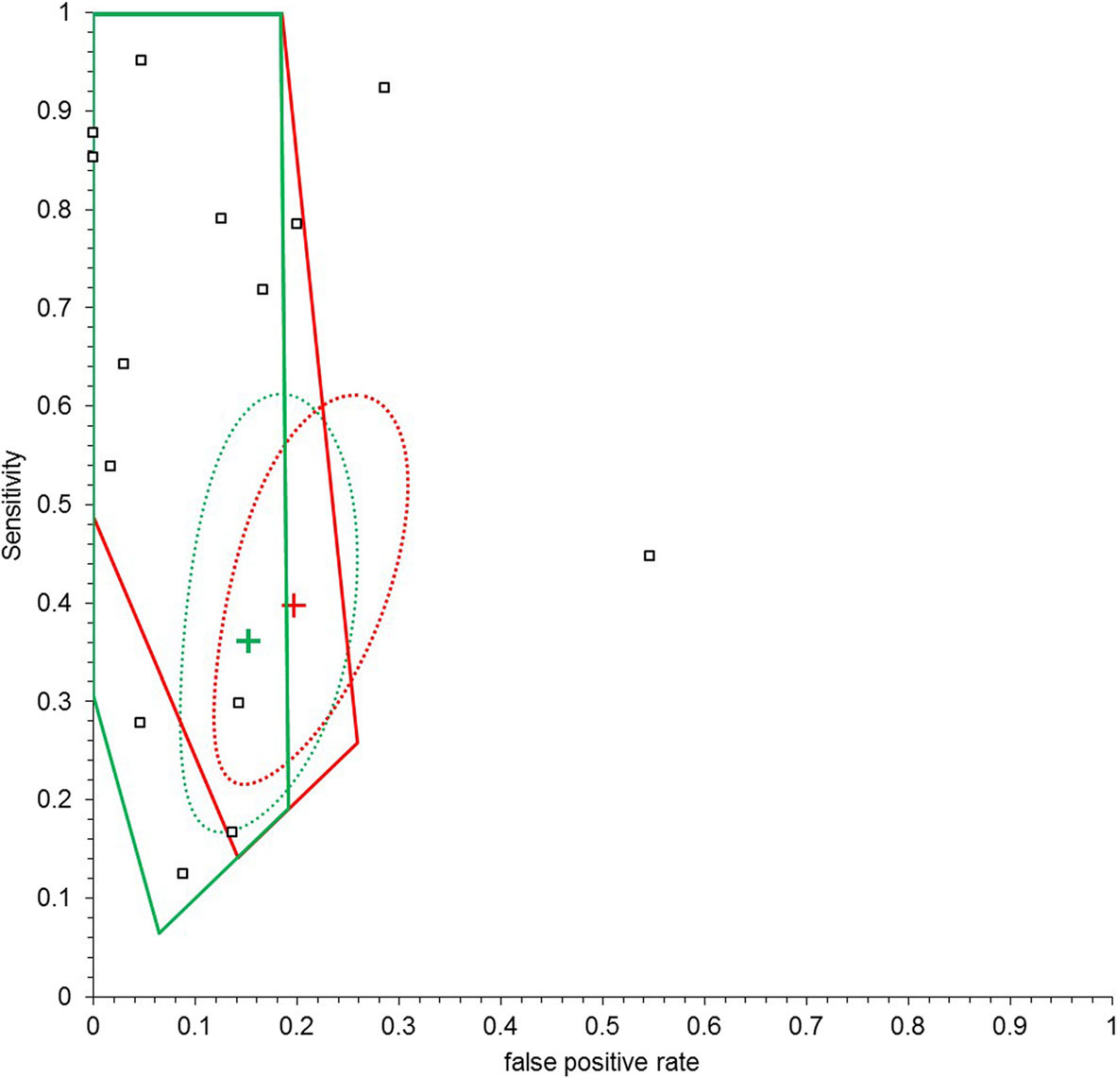
Comparison of tailored meta-analyses for the radiographic detection of all occlusal lesions for Egypt (red) and Germany (green). Also given are the summary estimates (cross) with the associated 95% confidence ellipses

**Fig. 2:**
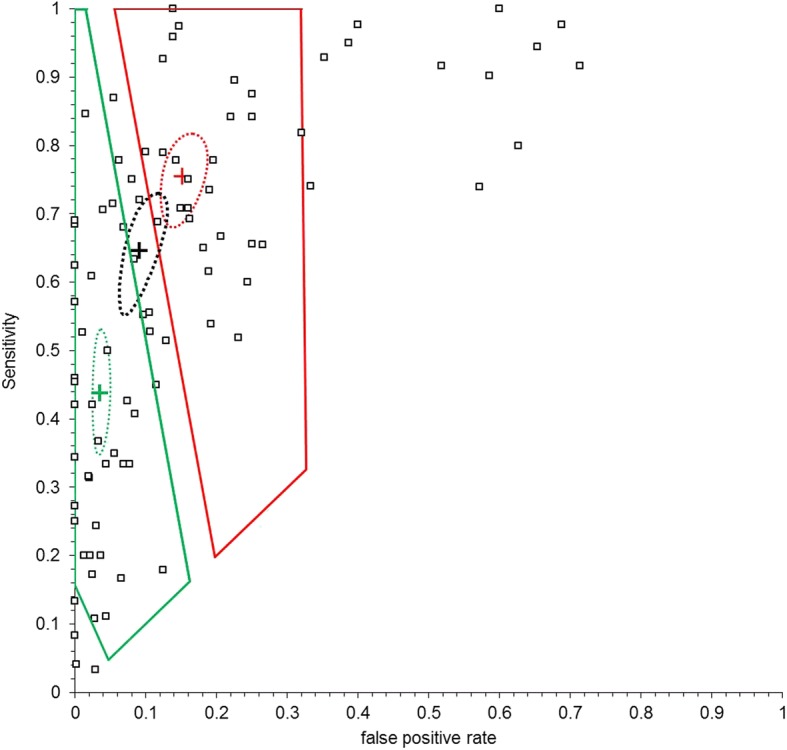
Comparison of tailored meta-analyses for the visual detection of advanced occlusal lesions for Egypt (red), Germany (green) and the conventional meta-analysis (black). Also given are the summary estimates (cross) with the associated 95% confidence ellipses

From the original reviews [3, 6], 210 studies on visual caries detection and 100 studies on radiographic caries detection were included in our meta-analyses (see Additional file 1). Inspection of the ROC plots for the conventional meta-analyses demonstrates widespread dispersion of studies, suggesting that heterogeneity is present in all the analyses as expected. Figures 1 and 2 illustrate this for the radiographic detection of occlusal lesions and the visual detection of advanced occlusal lesions.

From the routine data interval estimates for the test positive rate and prevalence were calculated (Table 1) and these were used to derive an applicable region [10, 11] for each of the tests in Egypt and Germany. This is illustrated for the radiographic detection of occlusal lesions and the visual detection of advanced occlusal lesions in Figs. 1 and 2.

In Fig. 1 the applicable regions for Egypt and Germany are close and overlap in large areas of the ROC space, whereas in Fig. 2 they occupy discrete regions. Thus, the visual detection of advance occlusal lesions in Egypt is likely to have sensitivity and specificity which is in a completely different region of ROC space from that of Germany. Furthermore, from Fig. 2 the conventional point estimate (all studies included) lies between the applicable regions for both Egypt and Germany and is significantly different from the tailored estimate for Germany.

The location of the applicable region in ROC space affects the number of studies included in the tailored meta-analyses and in general, tailoring to the setting reduces the number of studies applicable (Table 2). Thus for the visual detection of all occlusal lesions out of the 67 studies included in the original meta-analysis only 51 studies were applicable to Egypt and only 12 studies were applicable to Germany. This can have profound effects on the estimates for the sensitivity and specificity which are plausible for these two settings.

**Table 2:** Mean (95% CI) sensitivity and specificity for all studies, tailored to Egypt and tailored to Germany

	ALL			Egypt			Germany		
	n	Sensitivity	Specificity	n	Sensitivity	Specificity	n	Sensitivity	Specificity
Visual detection									
advanced occlusal	94	64.6% (57–71)	90.9% (88–93)	55	75.1% (70–81)	84.9% (82–89)	50	43.7% (37–51)	96.5% (95–97)
all occlusal	67	85.5% (81–89)	75.3% (70–80)	51	85.8% (82–89)	77.0% (73–81)	12	49.1% (40–58)	91.2% (88–94)
Radiographic detection									
advanced occlusal	44	52.3% (44–60)	89.3% (86–92)	38	54.2% (47–61)	89.1% (86–92)	36	50.8% (43–59)	91.4% (88–94)
all occlusal	14	38.6% (25–54)	77.8% (70–84)	11	39.7% (25–56)	80.4% (72–87)	9	36.0% (20–56)	84.8% (77–90)
advanced proximal	22	44.4% (38–51)	95.5% (94–97)	22	44.4% (38–51)	95.5% (94–97)	22	44.4% (38–51)	95.5% (94–97)
all proximal	38	43.2% (36–51)	90.0% (87–92)	31	40.2% (32–48)	92.5% (91–94)	34	40.5% (33–48)	91.7% (90–93)

n = number of studies

Table 3 illustrates the effects tailoring has on the likelihood ratios for the test. The most marked differences occur with the visual detection of occlusal lesions. In contrast, radiographic detection is broadly consistent across both settings (Egypt and Germany) and in line with the average estimates when all studies are included.

**Table 3:** Mean (95% CI) Positive and Negative Likelihood Ratios (LR) for all studies, tailored to Egypt and tailored to Germany

	ALL			Egypt			Germany		
	n	Positive LR	Negative LR	n	Positive LR	Negative LR	n	Positive LR	Negative LR
Visual detection									
advanced occlusal	94	7.09 (5.6–8.9)	0.39 (0.3–0.5)	55	5.0 (4.3–5.8)	0.29 (0.2–0.4)	50	12.5 (9.2–16.9)	0.58 (0.5–0.7)
all occlusal	67	3.46 (2.8–4.3)	0.19 (0.15–0.25)	51	3.73 (3.1–4.4)	0.18 (0.14–0.24)	12	5.56 (3.7–8.5)	0.56 (0.46–0.68)
Radiographic detection									
advanced occlusal	44	4.90 (3.7–6.5)	0.53 (0.46–0.62)	38	5.96 (3.7–6.6)	0.51 (0.44–0.60)	36	5.87 (4.3–8.0)	0.54 (0.46–0.63)
all occlusal	14	1.74 (1.3–2.4)	0.79 (0.65–0.96)	11	2.02 (1.4–2.9)	0.75 (0.60–0.94)	9	2.36 (1.4–4.1)	0.76 (0.58–0.99)
advanced proximal	22	9.82 (7.3–13.3)	0.58 (0.52–0.65)	22	9.82 (7.3–13.3)	0.58 (0.52–0.65)	22	9.82 (7.3–13.3)	0.58 (0.52–0.65)
all proximal	38	4.30 (3.4–5.5)	0.63 (0.56–0.72)	31	5.34 (4.2–6.8)	0.65 (0.57–0.74)	34	4.89 (3.9–6.2)	0.65 (0.57–0.73)

The positive and negative predictive values (PPV, NPV) for the tests are given in Tables 4 and 5 using the point prevalence estimates for the settings where available. Again the differences between Egypt and Germany are large for visual detection and are driven by the differences in the prevalence and likelihood ratios. A PPV of 82% for visual detection of all occlusal lesions in the German setting suggests it should be used for ruling in occlusal lesions when the test is positive, whilst in Egypt the same test performs with a PPV of 36%. In contrast, a negative test result is more useful to clinicians in Egypt (NPV = 97%) compared with an NPV of 69% in the German setting.

**Table 4:** Positive Predictive Values (PPV) of different tests for Egypt and Germany where prevalence rates are available

	Egypt			Germany		
	Prevalence	Positive LR	PPV	Prevalence	Positive LR	PPV
Visual detection						
advanced occlusal	8.1%	5.00	30.6%	22.0%	12.5	77.9%
all occlusal	12.9%	3.73	35.6%	45.0%	5.56	82.0%
Radiographic detection						
advanced occlusal	19.0%	5.96	58.3%	10.0%	5.87	39.5%
all occlusal	19.0%	2.02	32.1%	11.0%	2.36	22.6%

**Table 5:** Negative Predictive Values (NPV) of different tests for Egypt and Germany where prevalence rates are available

	Egypt			Germany		
	Prevalence	Negative LR	NPV	Prevalence	Negative LR	NPV
Visual detection						
advanced occlusal	8.1%	0.29	97.5%	22.0%	0.58	85.9%
all occlusal	12.9%	0.18	97.4%	45.0%	0.56	68.6%
Radiographic detection	19.0%	0.51	89.3%	10.0%	0.54	94.3%
advanced occlusal	19.0%	0.75	85.0%	11.0%	0.76	91.4%
all occlusal	8.1%	0.29	97.5%	22.0%	0.58	85.9%

## Discussion

When identifying carious lesions, general dental practitioners are faced with a specific patient population, where the prevalence rates of different lesions (and stages) are not always represented by those obtained from individual studies. Moreover, each practitioner will apply methods differently, yielding different test positive rates in the hands of different dentists. As a result, the PPV and/or NPV from a DAS are possibly very different from those found in a specific practice. The resulting over- and under-detection have implications both on a clinical and a public health level [4, 5].

When deciding if and how to apply a caries detection method, dentists should be cautious not to solely rely on the aggregate estimates from conventional diagnostic test meta-analyses particularly if there is widespread heterogeneity, as the implicit assumption of ‘one-size fits all’ may be untenable. The heterogeneity [19] stemming from factors like study design, test execution, threshold and patient spectrum can be generally described. However, it is difficult to determine which factors are responsible for the result of heterogeneity between DAS, and these cannot be addressed by conventional meta-analysis. Tailored meta-analysis circumvents this by using information directly from the setting of interest to determine which sub-group of studies are likely to reflect the performance in that setting.

We used tailored meta-analysis to assess whether the summary estimate of all DAS included in conventional meta-analysis is accurate in a setting such as Germany or Egypt. Including setting-specific test positive rates and prevalence allowed us to tailor our diagnostic meta-analysis. We showed that for visual detection of occlusal lesions the tailored meta-analysis estimates for Egypt and Germany do not coincide whereas for radiographic detection they do. This may have a number of reasons. First, test conduct may differ, starting from who performed the examinations (both visual and radiograpically) and how exactly this was done. Obviously, these aspects will not be 100% standardized even under controlled settings, and it is impossible to standardize them across settings in routine care. Tailoring meta-analysis can be useful here to yield setting-specific estimates which are more applicable under the specific circumstances. Second, prevalence rates differed, as described, reflecting different health conditions and risk factors (diet, availability of fluoride, oral hygiene), but also test positive rates. For example, we only evaluated accessible surfaces, which usually meant for occlusal surfaces, unsealed ones. In Germany, unsealed surfaces are found mainly in patients with irregular utilization of dental services; these patients usually also show high caries risk [12]. Low risk patients usually attend the dentist regularly; the majority of occlusal surfaces in these surfaces are sealed for preventive reasons [12]. The resulting high test positive rate for caries lesions in the available surfaces is a result of this; consequently, visual detection had high specificity and PPV, but relatively low NPV. Hence, on occlusal surfaces, dentists in this specific German setting can expect positive detections of caries on occlusal surfaces to be true, and treat accordingly. Negative detections, in contrast, may be false; an additional (more sensitive) diagnostic measure could be applied additionally to increase the NPV. In contrast, in Egypt the tooth level prevalence of occlusal lesions was very low, resulting in low specificity and low PPV of visual detection. Hence, positive detections should be regarded with caution and an additional test for verifying the positive test result should be considered prior to applying (invasive) treatments.

In contrast, for radiographic assessments, tailored meta-analysis did not yield significantly different findings for both settings or compared to conventional meta-analysis. This may have a number of reasons, too. First, we did not tailor for prevalence of proximal lesions; this was, as no epidemiologic data for both settings was available. Hence, our estimates for radiographic detection are not as tailored as those for visual detection. Second, prevalence rates may indeed be similar in both settings (something we don’t know) for proximal lesions. Third, it can be assumed that interpreting radiographs is -to some degree- more objective than visual assessment; the impact of test conduct may be lower for radiographic than for visual detection. Fourth, heterogeneity was generally lower for DSA on radiographic detection; the impact of tailoring will automatically be lower under such circumstances. Last, the number of studies included in this meta-analysis was lower; confidence intervals for any accuracy estimates were wider due to lower statistical power and differences between settings less likely to be detected.

### Limitations

The main driver of tailored meta-analysis is the interval estimate for the test positive rate which determines the size of the applicable region. The interval estimate for the prevalence helps refine the applicable region further. Thus the estimates may be subject to selection bias. This was mitigated by sampling consecutive patients although it is still possible that an unrepresentative sample was selected.

Only certain surfaces on molars were assessed as both visual occlusal and radiographic proximal assessment are useful detection methods here. In practice, dentists would assess other teeth and surfaces, too. This may affect the prevalence rates reported in this study.

Many of the primary studies were conducted in vitro and showed high risk of bias, often due to unrealistically high prevalence of lesions and potential spectrum bias. However, when there are multiple sources of heterogeneity the individual contributions of the different sources of heterogeneity are unlikely to be determined. In such an instance, tailored meta-analysis may afford the advantage of being probabilistic rather than deterministic in its study selection.

Some surfaces were not accessible for inspection due to teeth not being fully erupted, teeth overlapping on radiographs, presence of sealants or presence of restorations. The presence of a restoration indicates a previous lesion on the surface and omitting such cases from the data could potentially lead to the test positive rate being underestimated. However, there were only a few such cases and their effect on our estimates is likely to be limited.

### Recommendations

Diagnostic accuracy studies should be scrutinized for their applicability to different settings, and a critical risk of bias assessment with a focus on setting-specific prevalence and patient spectrum should be performed. A systematic assessment of the risk of bias may be made using tools such as QUADAS-2 [20] prior to the study conduct to allow a higher internal validity.

It is clear from this analysis and others [10, 11] that the summary estimates yielded from conventional meta-analysis of diagnostic accuracy studies have the potential to be inapplicable in particular clinical settings and tailored meta-analysis potentially overcomes this. Although at presence it remains the reserve of statisticians, a user-friendly package in which medical practitioners could readily implement it in the clinical setting would widen the access of the technique.

One of the difficulties for systematic reviewers is the inadequate reporting of data in the primary studies. The STARD statement, which provides standards on reporting of primary diagnostic studies, aimed to address this [21]. Although any improvements in reporting remain largely the responsibility of primary research investigators, journals have a role in facilitating this process and ensuring the STARD statement is fully observed in published diagnostic test accuracy studies.

Although tailored meta-analysis produces estimates which are plausible, their validity is yet to be established. Recently there have been developments in the evaluation of the validity of meta-analysis estimates [22]. Potentially such methods could be used to investigate the validity of tailored meta-analysis findings, but at present this an area for future research.

Existing or newly developed guidelines on caries detection methods should include not only generic, but tailored accuracy estimates, where possible, before deciding to recommend or refute the application of a detection method. Routinely collected data (which is increasingly available) might be useful for such tailoring. In the long term, tailoring might be performed using routine data via practice software, with generated outputs assisting practitioners in individualized decision-making.

## Conclusions

Conventional test accuracy meta-analyses may yield aggregate estimates which are inapplicable to specific settings due to the failure of the summary estimate to capture the variation in test performance across different settings. However, routine data collected from the setting of interest may be combined with secondary research to modify this estimate to produce an estimate which is tailored to the setting. This estimate is more likely to be representative of the test accuracy within the setting of interest. Test accuracy meta-analyses should be scrutinized for their applicability to different settings and tailoring may eventually be useful for individualized decision-making.

## Additional file

Additional file 1: All data used the in the meta-analysis, tabulated. (ZIP 105 kb)

## Abbreviations

DAS: Diagnostic accuracy study
ICDAS: International Caries Detection and Assessment System
MLE: Maximum likelihood estimate
NPV: Negative predictive value
PPV: Positive predictive value
ROC: Receiver Operating Characteristics
TMA: Tailored Meta-Analysis
